# Cartas al editor

**Published:** 2020-11-15

Pereira, 15 de octubre de 2020

Señores Comité Editorial Revista *Biomédica*

Estimados señores:

Después de leer el artículo "Evaluación comparativa de la vigilancia en salud pública de COVID-19 en Colombia: primer semestre" ^1^, cabe resaltar el interés de la evaluación del sistema de vigilancia epidemiológica en el marco de esta pandemia. Se constata el efecto positivo que la inyección de recursos de todo tipo ha tenido en el sistema en la transformación de las condiciones previas y la superación de inequidades.

Los autores plantean en su artículo que la evaluación se basó en el criterio de cumplimiento de la ley de Benford. Quisiéramos señalar que no conocíamos dicha ley, y que no encontramos ninguna validación de sus postulados en el artículo publicado, ya que solo se alude en él a su aplicación por parte del autor de correspondencia en otros estudios suyos. En nuestra búsqueda en la literatura especializada encontramos que en el artículo de Cerioli, *et al.*^2^, los autores señalan que "[...] ninguna teoría general conocida puede predecir con exactitud si la ley de Benford es válida en una aplicación específica, cuyo proceso de generación de datos no se puede conocer con certeza, incluso en ausencia de fraude u otras manipulaciones de datos" (traducción libre). Cerioli, *et al.* citan, a su vez, a Berger, *et al.*^3^, quienes advierten que la ley de Benford "[...] no parece proporcionar información. Un fenómeno amplio y a menudo mal entendido no siempre debe reducirse a unos pocos modelos matemáticos" (traducción libre). A renglón seguido los autores añaden que "[...] actualmente no existe un enfoque unificado que explique simultáneamente la aparición de un fenómeno ni en sistemas dinámicos, teoría de números, estadísticas y datos del mundo real" (traducción libre). En otro artículo ^4^ se afirma que la ley de Benford asume distribuciones normales para modelar los componentes aleatorios en sus conteos simulados, pero si los segundos dígitos siguen una distribución normal, en general no satisfacen la ley de Benford. Además, no hay razón para creer que los datos simulados se parecen a los datos registrados en cualquier epidemia.

Por otra parte, como resultado de la revisión bibliográfíca que hicimos, supimos que la ley de Benford exige el supuesto de independencia estadística de los dígitos, ante lo que surge la pregunta de cómo evaluaron este supuesto los autores, especialmente en una situación de pandemia en la cual es difícil establecer la independencia de un caso con respecto a otro, sobre todo porque se trata de una infección que se transmite entre personas, y estas no son independientes unas de otras.

Los autores aluden desde el resumen que determinaron los departamentos con un desempeño óptimo, pero no queda claro en qué consiste dicho desempeño, aunque, al parecer, tiene que ver con el cumplimiento de la ley de Benford para el primero y segundo dígitos. En la introducción hacen el recuento juicioso de algunas características de la pandemia en Colombia, resaltando las diferencias de la epidemia según regiones, pero posteriormente hacen comentarios con base en criterios no aclarados: ¿cuál es la definición de pico?, ¿cuál es el procedimiento y el análisis para determinar el pico? Mencionamos esto porque hay mucha discusión al respecto, por ejemplo, hay autores que han planteado que nos equivocamos al pensar que Europa había salido del ascenso en agosto, lo que resulta interesante en sí mismo por el aumento en la frecuencia de casos asociados con la tasa de transmisión y la capacidad de los servicios de salud para detectar y notificarlos. Pero, ¿realmente cuándo llegaremos nosotros al pico? ¿No será que este ocurre al llegar a un número muy cercano al 50 % de infección? ¿Sabemos cuál es la prevalencia de la infección en el país? Lo que los autores denominan "pico" es realmente un número máximo de casos a nivel regional, y con base en ese número de casos acumulados se comparan los departamentos. La pregunta que nos hacemos es, ¿son comparables nuestros departamentos? Es decir, ¿estamos en el mismo nivel de desarrollo de la pandemia? ¿Están todas las regiones en igualdad de condiciones para detectar, aislar y notificar los casos? Si no es así, ¿saben los autores qué están comparando al cotejar los datos de los departamentos? ¿Qué quiere decir la meseta en la frecuencia de casos? ¿Se estabilizó la tasa de transmisión y la capacidad diagnóstica, y se saturó el reporte de casos en la vigilancia epidemiológica? ¿Cuál es la utilidad de una métrica cuando lo que voy a medir no se encuentra bajo la misma condición exigida por esta? En el texto también hay algunas declaraciones que deben definirse porque son parte de la evaluación, por ejemplo, ¿qué es la calidad del dato?, ¿bajo qué reglas se estandariza la evaluación comparativa?

Nosotros hicimos una simulación de la ley de Benford y la conclusión fue que cuando el crecimiento es modelado de forma exponencial, se cumple con la ley y con lo tocante al primer digito, y si esto ocurre, no hay contención, pero los autores del artículo claramente afirman lo contrario. Con base en la simulación que hicimos concluimos que el virus se diseminó exponencialmente y que el plan de contención fracasó. En este sentido, cumplir la ley y lo relacionado con el primer digito simplemente permite reportar un número de casos en relación con el crecimiento exponencial, y eso no es algo para destacar, sobre todo cuando el fracaso de la contención se propone como criterio de "desempeño" de las labores de vigilancia en salud pública.

Además, los autores plantean una falacia lógica cuando, bajo un razonamiento aparentemente correcto, llegan a una conclusión errónea afirmando que el desempeño es óptimo. Es un error afirmar que el número de casos iniciados afecta el cumplimiento de lo concerniente al segundo digito, dado que, según la ley de Benford, para ello es necesario tener una muy alta tasa de transmisión. Solo de esa manera aumentan las probabilidades de cumplir con ella en cuanto al segundo digito. ¿Esto es buen desempeño? Evidentemente no lo es, pues lo que esto sugiere es que el plan de contención fracasó y que la tasa de transmisión nunca logró disminuirse.

Solo con propósitos ilustrativos los invitamos a revisar la simulación de la ley de Benford; en ella se pueden observar las consecuencias de dicha ley en el número de casos positivos reportados en el tiempo.

En epidemiologia nos interesa disminuir la tasa de transmisión para aplanar la curva que describe el número de nuevos casos en el tiempo. Cumplir con la ley de Benford no necesariamente es un criterio adecuado para evaluar la calidad de la vigilancia epidemiológica y de las intervenciones. Los departamentos que no cumplen con los postulados de la ley pueden aplanar la curva si la epidemia se comporta como lo definen estos.

Sin ninguna argumentación, los autores generan una clasificación con base en un procedimiento que no es claro, lo que plantea preguntas sobre lo que significa dicha clasificación, lo que mide, si es deseable tener un desempeño bueno o malo según ella, y si es posible sacar conclusiones o derivar mecanismos para mejorar el "desempeño" y la posición en la clasificación. Por ejemplo, según el artículo ¿qué deben hacer las regiones para mejorar su posición en dicha clasificación? Si esta es una escala, ¡qué sensibilidad y especificidad tiene? ¿Qué replicabilidad?

Realmente, el artículo incluye muchos juicios de valor, pues no se definen aspectos críticos de la evaluación y la comparación. Tampoco es riguroso en la adaptación y la aplicación del modelo matemático a la situación planteada y, peor aún, no es claro cuál es el problema ni la pregunta de investigación. Simplemente se plantea una hipótesis cuya validez nunca es evaluada ni contrastada con otros métodos. El artículo tampoco es claro en manifestar qué evalúa y no define un plan de mejoramiento para cada una de las regiones en función de sus particularidades, con lo que se pierde la posibilidad de aportar planteamientos técnicos y científicos para un mejor desempeño frente a la pandemia.

Atentamente,

José William Martínez

Facultad de Ciencias de la Salud, Universidad Tecnológica de Pereira, Pereira, Colombia

Juan Camilo Martínez 

Universidad de Vanderbilt, TN, USA

Diego Alejandro Rincón, Diego Alejandro Salazar, Juan Daniel Castrillón, María del Pilar Gómez, Oscar Felipe Suárez Brochero, Juan Pablo Vélez, Ángela María Valencia 

Secretaría de Salud de Risaralda, Pereira, Colombia

Sandra Gómez

Dirección Operativa de Salud Pública de Risaralda, Pereira, Colombia Ángel María Rincón Hurtado

Fundación Universitaria del Área Andina, Pereira, Colombia 
