# Supplementary material for: Cartas al editor
Source: Biomedica. 2020 Nov 15;40(Suppl 2):198–204. [Article in Spanish] (PMC7676834)
Supplement: Related texts

# Reply: Cartas al editor

Bogotá, 15 de agosto de 2020

Señores Comité Editorial Revista *Biomédica*

Estimados señores:

Hemos leído la carta enviada por Martínez, *et al.,* en la que se discute nuestro artículo "Evaluación comparativa de la vigilancia en salud pública de COVID-19 en Colombia: primer semestre" ^1^. Dado que los mismos autores reconocen que son neófitos en el tema de la ley de Benford y su aplicación en la vigilancia en salud pública, responderemos a sus inquietudes, las cuales coinciden con las que algunos de nosotros tuvimos hace unos años cuando abordamos esta temática de manera sistemática y propusimos su uso para evaluar sistemas de vigilancia en salud pública en el contexto de las epidemias. Esa primera aproximación quedó plasmada en un artículo publicado en el 2011 ^2^, que se centraba en el modelado matemático de epidemias de infecciones emergentes. En esa ocasión, los revisores y editores nos brindaron aportes valiosos que incorporamos en la versión del artículo que fue publicado en dicho momento.

Ante todo, nuestro aporte no apunta a los sistemas de vigilancia epidemiológica, como lo afirman los colegas en su carta, sino a los sistemas de vigilancia en salud pública, concepto diferente que no entraremos a discutir, pues ya ha sido descrito por otros autores ^3^^,^^4^.

En segundo lugar, la evaluación de este tipo de sistemas incluye muchos elementos, pero nuestro enfoque se centró exclusivamente en la calidad de los datos, el cual es uno de los atributos que diversas agencias internacionales especializadas en esta tarea reconocen como importantes. Los otros atributos son la simplicidad, la flexibilidad, la aceptabilidad, la sensibilidad, el valor predictivo positivo, la representatividad, la oportunidad y la estabilidad ^5^. En ese sentido, nuestra contribución se centró en la evaluación rápida y objetiva de un solo atributo, sin sesgos ni conflictos de intereses, y se basa en premisas que cualquiera que acceda a los datos puede replicar, lo que es posible por la política de datos abiertos de la pandemia. Nuestro artículo presenta los datos de tal forma que, incluso, pueden interpretarse con un algoritmo diferente al usado por nosotros. Entendemos que la calidad de los datos es un atributo de gran importancia. Por ejemplo, no es un buen sistema de vigilancia en salud pública aquel que, aunque tenga elevadas sensibilidad y oportunidad, falle en cuanto a la calidad de los datos; por ello, el desempeño del sistema sí tiene relación con el cumplimiento o no de la ley de Benford.

Tercero, la presentación de casos durante una pandemia es difícil de determinar con las estrategias estándar de la vigilancia en salud pública. Sin embargo, mediante conceptos como el R_0_ y los modelos de transmisión, los fundamentos de la epidemiología de las enfermedades infecciosas indican que el comportamiento teórico esperado en lo que respecta a los casos acumulados sigue un patrón relacionado. Este hecho explica que se obtengan más números cuyo primer dígito sea el uno, seguido por el dos, luego el tres y así sucesivamente hasta el nueve. Estos datos esperados, cuya distribución se define por la ley de Benford, son los que se contrastan, mediante pruebas estadísticas, con los datos observados resultantes del proceso de vigilancia en salud pública. En varias de las referencias de la presente respuesta se encuentran diversos usos y explicaciones más detalladas ^2^^,^^6^^-^^10^.

En cuarto lugar, la suposición estadística de independencia pudo ser mal interpretada por los colegas. Dado el diseño, se espera que ningún caso de COVID-19 ocurra independientemente de otro debido a las características propias de la transmisión, y el análisis basado en la ley de Benford que presentamos en nuestro artículo, evalúa la frecuencia de los dígitos de los reportes de casos, no los casos en sí, por lo que no presupone la independencia de los casos ni afecta los métodos empleados. En nuestro artículo se usa la ley de Benford para verificar si al sumarse los casos en una ventana de tiempo definido, la cantidad de casos acumulados notificados (primer o segundo dígito) ocurre sin adoptar un patrón diferente al orden esperado en el supuesto de que no se dé una intervención o no se logren las cifras. La independencia en este caso depende de que no haya modificaciones en la información que provoquen frecuencias alteradas de casos, no al hecho de que un caso intervenga en la aparición de otro, que es lo natural y esperado.

En quinto lugar, es importante señalar que no era el interés del artículo la validación de la ley de Benford para su uso en la vigilancia en salud pública. Los procesos epidemiológicos de validación no atañen a la verificación propuesta en este trabajo en torno a la idoneidad de las cifras provenientes de esa vigilancia y su cumplimiento de la ley de Benford; en su lugar, la validación se sitúa en la necesidad de valorar la adaptabilidad y capacidad con la que un sistema *ad hoc* para la medición o evaluación de un constructo sanitario funciona en distintos contextos o circunstancias ^13^. Sin embargo, consideramos que la validación del método debería hacerse mediante su contraste con la realidad que observamos. Si bien las experiencias previas con la influenza A(H_1_N_1_), el dengue y el Zika nos dieron algunos elementos, la pandemia de COVID-19 ha sido un mejor escenario para probar el método debido la amplia divulgación de datos y las noticias de dominio público.

En los primeros momentos de la pandemia nos surgió una inquietud en torno a la calidad de los datos de la epidemia en la República Popular China, por lo que se aplicó el método y evidenciamos que estos cumplían con la ley de Benford ^6^. Este hallazgo fue reportado con análisis similares por varios autores de diversas instituciones y países en más de 10 artículos y publicaciones anticipadas ^8^^-^^11^, concordancia de resultados que evidencia la validez del enfoque analítico. Un caso cercano e interesante es Brasil, cuyo presidente se ha mostrado negacionista frente a la pandemia, y en donde hay evidencias sobre los cambios arbitrarios introducidos en la manera de informar los casos de infección por SARS-CoV-2 y las muertes asociadas. En este caso, el empleo de la ley de Benford de forma similar a la nuestra, permitió establecer que los datos entre julio y agosto sobre muertes y casos positivos, no cumplieron con los criterios de calidad ^12^. En el caso de Colombia, el que los datos de algunos departamentos no cumplieran con la ley de Benford podría relacionarse más con rezagos en la detección o captación de los casos.

En sexto lugar, la capacidad del artículo de mejorar el desempeño de la vigilancia en salud pública escapaba de su objetivo. Lo que sugerimos es que se revisen los casos que cumplieron con la ley de Benford para determinar en qué áreas podría mejorarse. Si son exitosas, las intervenciones y las acciones sanitarias que se desplieguen en cada territorio tendrán como uno de sus efectos, no el único, mejorar la calidad de los datos y, en consecuencia, habrá también mejoras en el cumplimiento de la ley de Benford. En este sentido, podemos afirmar que es más difícil cumplir con lo tocante a los primeros dígitos (1, 2 y 3) que a los últimos (7, 8 y 9). Aunque el estudio se centró en contribuir a conocer la situación nacional de uno de los atributos de los procesos de vigilancia en salud pública, es decir, la calidad de los datos, el cumplimiento o no de la ley de Benford no descalifica ni demerita el esfuerzo de nadie, y la clasificación de los departamentos da cuenta de ello; las afirmaciones que se desprenden de este resultado podrían evidenciar las dificultades para interpretar, específicamente, la calidad de los datos.

En séptimo lugar, al referirse a las falacias, los autores de la carta al editor deberían señalar con más precisión a cuál aluden. Los "juicios de valor" como los llaman los autores, apenas son conclusiones derivadas del análisis hecho a partir de la implementación del método. Vemos la controversia que nuestro estudio suscitó como un paso más hacia una mejor comprensión del funcionamiento de los sistemas de vigilancia en salud pública, de hecho, ¡creemos que ese es precisamente su objetivo!

Por último, nuestra experiencia de más de 10 años trabajando con la ley de Benford aplicada a los sistemas de vigilancia en salud pública, nos indica que cuando no se cumple es importante que se revise si hace falta la detección de un número mayor de casos o su registro adecuado. Además, en este caso debería incorporarse todo lo relacionado con la capacidad de los laboratorios de salud pública en este análisis, dada su dependencia para diagnosticar los casos. El propósito de nuestro estudio no fue evaluar la respuesta a la pandemia en el nivel territorial si no valorar la calidad de los datos captados por los sistemas de vigilancia a nivel local utilizando la Ley de Benford.

Álvaro J. Idrovo, Alexandra Hurtado-Ortiz

Departamento de Salud Pública, Escuela de Medicina, Universidad Industrial de Santander, Bucaramanga, Colombia

José Moreno-Montoya

Subdirección de Estudios Clínicos, Fundación Santa Fe de Bogotá, Bogotá, D.C., Colombia Franklyn E. Prieto-Alvarado

Dirección de Vigilancia y Análisis del Riesgo en Salud Pública, Instituto Nacional de Salud, Bogotá, D.C., Colombia

## References

[1] Hurtado-Ortiz A, Moreno-Montoya J, Prieto-Alvarado FE, Idrovo AJ (2020). Evaluación comparativa de la vigilancia en salud pública de COVID-19 en Colombia: primer semestre. Biomédica.

[2] Idrovo AJ, Fernández-Niño JA, Bojórquez-Chapela I, Moreno-Montoya J (2011). Performance of public health surveillance systems during the influenza A(H1N1) pandemic in the Americas: Testing a new method based on Benford's law. Epidemiol Infect.

[3] Langmuir AD (1971). Evolution of the concept of surveillance in the United States. Proc R Soc Med.

[4] Declich S, Carter AO (1994). Public health surveillance: Historical origins, methods and evaluation. Bull World Health Organ.

[5] German RR, Lee LM, Horan JM, Milstein RL, Pertowski CA, Waller MN (2001). Updated guidelines for evaluating public health surveillance systems: Recommendations from the Guidelines Working Group. MMWR Recomm Rep.

[6] Idrovo AJ, Manrique-Hernández EF (2020). Data quality of Chinese surveillance of COVID-19: Objective analysis based on WHO's situation reports. Asia Pac J Public Health.

[7] Koesters N, McMenemy A, Belanger Y Simulating epidemics with a SIRD model and testing with Benford's law.

[8] Zhang J (2002). Testing case number of coronavirus disease 2019 in China with Newcomb-Benford law. arXiv.

[9] Isea R (2020). How valid are the reported cases of people infected with covid-19 in the world?. Int J Coronav.

[10] Lee KB, Han S, Jeong Y (2020). COVID-19, flattening the curve, and Benford's law. Physica A: Stat Mech Appl.

[11] Elba F (2008). Dati Covid 2019 e legge di Benford. arXiv.

[12] Idrovo AJ, Manrique-Hernández EF, Fernández-Niño JA (2020). Report from Bolsonaro's Brazil: The consequences of ignoring science. Int J Health Serv.

[13] Boateng GO, Neilands TB, Frongillo EA, Melgar-Quiñonez HR, Young SL (2018). Best practices for developing and validating scales for health, social, and behavioral research: A primer. Front Public Health.
